# Phylogeography of the insular populations of common octopus, *Octopus vulgaris* Cuvier, 1797, in the Atlantic Macaronesia

**DOI:** 10.1371/journal.pone.0230294

**Published:** 2020-03-19

**Authors:** Javier Quinteiro, Jorge Rodríguez-Castro, Manuel Rey-Méndez, Nieves González-Henríquez

**Affiliations:** 1 Molecular Systematics Laboratory, Department of Biochemistry and Molecular Biology, University Santiago de Compostela, A Coruña, Galicia, Spain; 2 BIOMOL Laboratory, Department of Biology, University of Las Palmas de Gran Canaria, Las Palmas de Gran Canaria, Spain; Natural History Museum of London, UNITED KINGDOM

## Abstract

Exploited, understudied populations of the common octopus, *Octopus vulgaris* Cuvier, 1797, occur in the northeastern Atlantic (NEA) throughout Macaronesia, comprising the Azores, Madeira and Canaries, and also the Cabo Verde archipelago. This octopus species, found from the intertidal to shallow continental-shelf waters, is largely sedentary, and the subject of intense, frequently unregulated fishing effort. We infer connectivity among insular populations of this octopus. Mitochondrial control region and COX1 sequence datasets reveal two highly divergent haplogroups (α and β) at similar frequencies, with opposing clinal distributions along the sampled latitudinal range. Haplogroups have different demographic and phylogeographic patterns, with origins related to the two last glacial maxima. *F*_ST_ values suggest a significant differentiation for most pairwise comparisons, including insular and continental samples, from the Galicia and Morocco coasts, with the exception of pairwise comparisons for samples from Madeira and the Canaries populations. Results indicate the existence of genetically differentiated octopus populations throughout the NEA. This emphasizes the importance of regulations by autonomous regional governments of the Azores, Madeira and the Canaries, for appropriate management of insular octopus stocks.

## Introduction

Oceanic islands are a source of diverse biogeographic paradigms, with those related to genetic isolation, population differentiation and speciation of terrestrial fauna and flora being particularly common [1]. In the northeastern Atlantic (NEA), the Macaronesia biogeographic region essentially comprises oceanic seamounts and the islands of the Azores, Madeira and the Canaries archipelagos [2], and sometimes the neighboring continental coast of the Iberian Peninsula and Africa, extending to the Cabo Verde archipelago. In the marine realm, genetic patterns are highly variable, and affected by diverse factors and processes. The open ocean and hydrographic structures and currents represent barriers to dispersal for invertebrates frequenting insular intertidal rocky habitat and the adjacent continental shelf [3–5]. This results in populations that are susceptible to demographic collapse. To the contrary, population connectivity is supported by a near continuum of suitable habitat for populations typical of continental coastal habitat [6]. Species-specific biological factors affecting dispersal potential are expected to be identical for both insular and coastal populations.

The common octopus, *Octopus vulgaris* Cuvier, 1797, represents a species complex, with *O*. *vulgaris sensu stricto* found in the NEA and Mediterranean Sea. Several allopatric, cryptic Atlantic putative species have been referred to as Type I, from Caribbean Sea and the Gulf of Mexico, Type 2 from the western South Atlantic along the Brazilian coast, and Type 3, mainly along the coast of South Africa [7]. One species within this complex, *O*. *insularis* [8], has recently been described from northern Brazil and remote islands of the mid- to South Atlantic. Taxonomic delineation has been supported by phylogenetic clades recovered from single mitochondrial COX1 gene data [9], although earlier mitochondrial analysis has suggested that the diverse Atlantic types and *O*. *vulgaris* (*s*.*s*.) are synonymous [10].

*Octopus vulgaris* (*s*.*s*.) occurs mainly in intertidal and shallow subtidal rocky or sandy habitat. During the day this species frequents dens, but at night it becomes more active, displaying relatively short-distance movements within a home range. Inshore reproductive migrations have been reported [11]. Semelparous reproduction involves multiple paternity [12] and production of 100,000–500,000 eggs, assembled in strings, maintained by a non-feeding female, which dies after the eggs hatch [11]. Planktonic paralarvae remain in the water column for weeks to months, determined by oceanographic and environmental conditions, and habitat availability [11]. In one wild Mediterranean population (Sardinia) where 50% of males and females mature at mantle lengths (ML) of 70 and 120 mm, respectively [13], age range based on beak-ring counting was considered to be 200–220 days (0.57 years). Comparable lifespan estimates of about one year have been reported for the Mediterranean [13], Canaries [14], northwestern Africa [15], Senegal coast [16], and the Atlantic coast of South Africa [17]. Maturation age estimation is also similar to *O*. *cyanea* Gray, 1849 [18].

The population dynamics of *O*. *vulgaris* are highly sensitive to environmental factors, particularly during the larval stage, but they are also affected by fishing activity [19]. Diverse species-specific fishery regulations and management areas exist along the Macaronesian region, confusing official fishery statistics. FAO (Food and Agriculture Organization) reports include Madeira and Azores catches with those from the Portuguese continental shelf, moreover this octopus is consumed locally without being reported in statistics [20]. Nevertheless, assessment based on catch data from the Fishery Committee for the Eastern Central Atlantic (CECAF) regulatory area, which includes Macaronesia, recommends a reduction or maintenance of fishing mortality along the three latitudinally established stocks [21].

The genetic structure of *O*. *vulgaris* populations in the NEA has been rarely studied. There have been no studies on island population connectivity for this species [22]. A microsatellite loci analysis [23] along the Atlantic coast of the Iberian Peninsula suggested a significant level of inter-population differentiation at distances exceeding 200 kms, which is consistent with an isolation-by-distance model [24].

Genetic studies have focused on *O*. *vulgaris* populations within the Mediterranean Sea. Significant population genetic structure was detected along the eastern Mediterranean coast, with haplotypes highly divergent from those found in Atlantic waters, with a correlation between genetic and geographic distances [25]. Significant structure was also detected within the central Mediterranean, with a break between western and eastern Mediterranean basins [26]. Recent studies on microsatellite genetic variation along the central-western Mediterranean have suggested a differentiation consistent with an island model of isolation [27]. On the South African coast, a dominant haplotype, found also on the Senegal coast, was identified as divergent from those in the northeastern Atlantic; a rare divergent haplotype likely associated with an invasive event was also identified [28].

The taxonomic status of octopuses captured in the NEA is investigated to determine their relationship with *O*. *vulgaris* (*s*.*s*.). The population structure of this species, which, in post-paralarval stages has limited dispersal and unusual reproductive behavior, that is subjected to intense, frequently unregulated, fishery effort along the Macaronesian coast, is analyzed. Patterns inferred for this species are related to biotic and abiotic process affecting marine invertebrates in general, to describe a phylogeographic scenario for the NEA oceanic insular environment. As a result, genetic patterns are relevant to delineation of units used in fisheries management.

## Materials and methods

**Fig 1:**
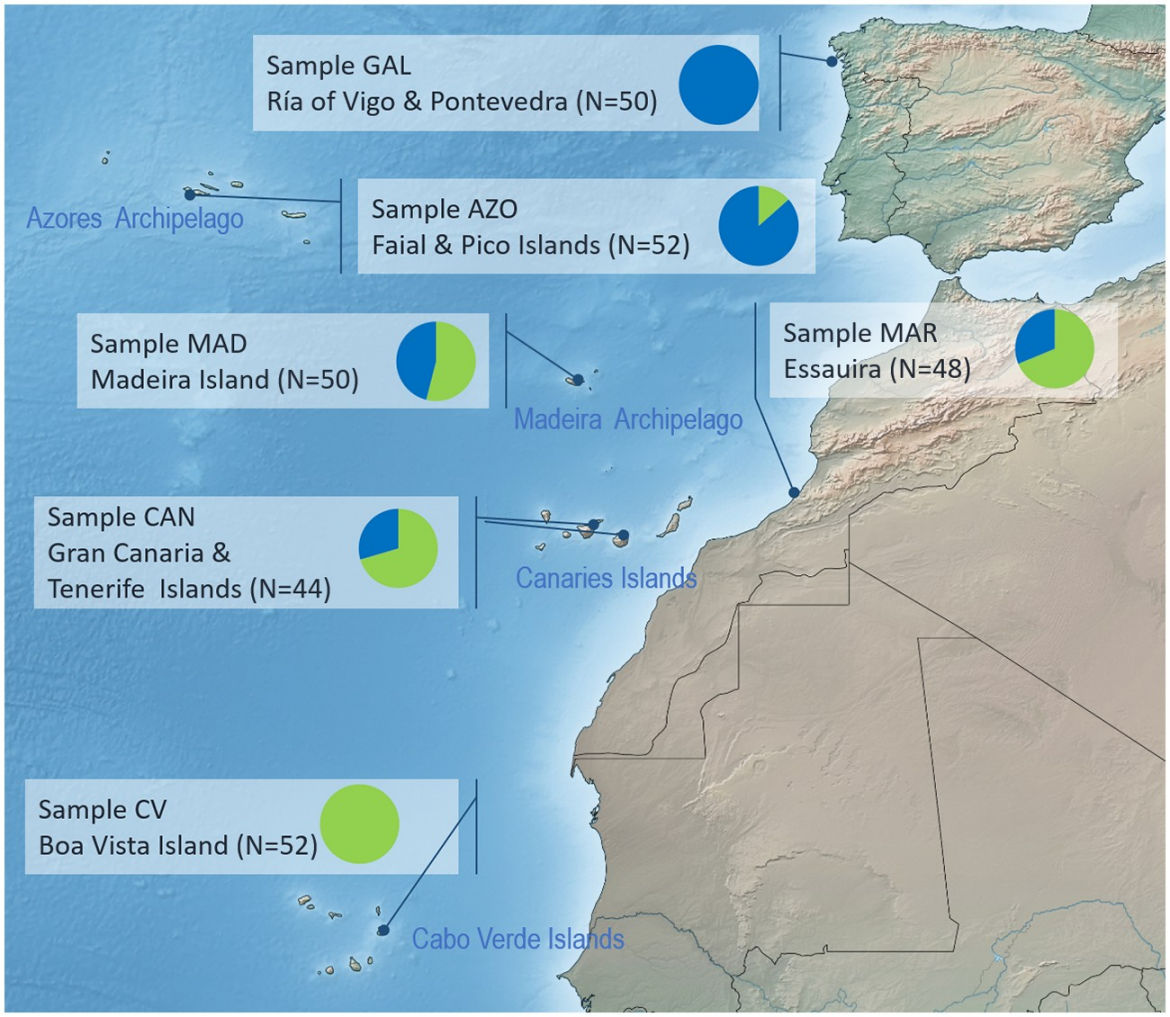
*Octopus vulgaris* sampling locations. Graphs depict proportions of specimens per site referable to one of haplogroup α (green) or β (blue), based on mitochondrial control region sequences. The map was generated using Simplemappr (www.simplemappr.net).

Samples comprising 296 specimens (44–52 individuals/sampling site) of *O*. *vulgaris* were obtained from six locations in the North Atlantic archipelagos of Azores, Madeira, Canaries and Cabo Verde, and two continental localities in Galicia (Northwest Iberian Peninsula, Spain) and the Atlantic Moroccan coast (Fig 1, Table 1). All analyzed specimens were caught as a result of artisanal, regulated and unregulated, fisheries activities. Muscle tissue samples were preserved in ethanol. DNA extraction was performed using a 30 mg sample of tissue and an E.Z.N.A.^®^ Mollusc DNA Kit (Omega Bio-tek; Norcross, Georgia, USA).

**Table 1:** Locations and summary statistics for *Octopus vulgaris* samples, including estimates of haplotype (*h*) and nucleotide (π) diversity, mismatch distribution parameters, neutrality, and demographic expansion test based on mitochondrial control region.

Sampling area	Lat. /Long.	N	Date	No. Hap.	*h*	π	τ	Tajima’s D	P	Fu’s Fs	P	Tau	Theta (θ) _0_	Theta (θ) _1_	H's R _i_	P	SSD	P
**Galicia**	42°20'/-08.88	50	2010	12	0.7861	0.0018	1.396	-1.56349	0.042	-6.6682	0.0001	1.396	0.001	99999	0.10132	0.074	0.00889	0.121
**Azores**	38°54'/-28.57	52	2010	13	0.6531	0.0097	0	0.13457	0.64	1.61851	0.756	0	0	99999	0.08998	1	0.51092	0
**Madeira**	32°63'/-16.91	50	2010	13	0.7845	0.0190	26.34	2.97211	0.99	6.33245	0.961	26.34	0	3.297	0.08792	0.399	0.11399	0.109
**Canaries**	28°06'/-16.03	44	2007/12	11	0.7072	0.0165	25.965	1.78177	0.979	6.10507	0.957	25.965	0	2.237	0.11287	0.45	0.09515	0.211
**Morocco**	31°41/-09.85	48	2012	17	0.8573	0.0199	28.289	1.74451	0.971	2.5078	0.836	28.289	0.002	24.464	0.06751	0.001	0.07090	0.027
**Cabo Verde**	16°06'/-23.07	52	2012/13	21	0.8771	0.0049	4.061	-0.85599	0.22	-10.69547	0	4.061	0.007	10.542	0.02803	0.56	0.00939	0.386

Two mitochondrial loci, a hypervariable and non-coding control region, and a 5’ partial segment of the COX1 gene, were analyzed. The complete mitochondrial control region of *O*. *vulgaris* was amplified using de novo designed primers OvulCR3F (5’-GAAAATCTTTCGTGCAAATTACACCACA-3’) and OvulCR4R (5’-TGTTAATGGTCAGGGTCTAAATTCAACTAAAT-3’), located in flanking tRNA-Glu and COX3 genes, respectively. The partial COX1 gene, homologous to the standard barcoding COX1 fragment, was amplified to obtain a complementary dataset using the new primers OvulCOX11F (5’-TGAATATTYTCAACAAATCAYAAAGAYATTGG-3’) and OvulCOX12R (5’-GGGTGACCAAARAATCAAAATARRTGTTG-3´). Amplification was performed in a representative sample of specimens (N = 42) harboring distinct hypervariable control region haplotypes which collapse in a reduced number of more conserved COX1 haplotypes.

Reactions were performed in a volume of 15 μL containing GoTaq Flexi Buffer (Promega, Madison, WI, USA), 1.5–3.5 mM MgCl_2_, 200 μM of dNTP, 0.5 μM of each primer, 0.15 units of GoTaq Flexi DNA Polymerase (Promega), and 10–50 ng of total DNA. PCR amplification protocol consisted of 95°C for 3 min, followed by 35 cycles including 95°C for 40 s, 60°C for 40 s, 72°C for 40s, then 72°C for 7 min, performed using a GeneAmp 9700 thermal cycler (Applied Biosystems).

PCR products were treated with Exo-SAP-It (Affymetrix, Santa Clara, CA, USA) to digest primers and deactivate unused dNTPs, and sequenced in both senses using a BigDye 3.1 sequencing kit (Applied Biosystems, Waltham, MA, USA). Extension products were purified with Montage SEQ 96 (Millipore) and separated and detected in a 3730xl Genetic Analyzer (Applied Biosystems). After chromatogram revision and trimming with Sequence Scanner (Applied Biosystems), sequences were aligned using Clustal X [29] implemented in BioEdit [30].

The basic haplotype (*h*) and nucleotide (π) diversity estimations, *F*_ST_ estimates for population differentiation, and diverse population parameters, including Tajima’s D and Fu’s *F*_*S*_, were obtained with Arlequin v.3.5 [31]. The haplotype network, depicting both the frequencies and relationship among detected haplotypes, was estimated using the median-joining algorithm in Network software [32]. Genetic differentiation across the sampled space, suggesting the number of panmictic groups and their limits, was assessed using a Geneland Bayesian program [33]. The number of most probable differentiated populations (*K; K* max = 5) was estimated after five multiple independent runs with 50,000 Markov Chain Monte Carlo (MCMC) iterations, with both correlated and uncorrelated allele frequency models. The hypothesis of an isolation-by distance scenario was tested with IBD and the Mantel test [34].

The model of DNA sequence evolution to best fit the data was selected using jModeltest v2.1 [35]. Molecular clock calibration by Bayesian criterion was performed using BEAST v.2.3.2 [36], based on the dating of closure of the Panama Isthmus [37] and recognized presence of geminate sister *Octopus* species clades within the Atlantic and Pacific oceans [38]. Geminate species were confirmed by phylogenetic inference using i) GenBank COX1 sequences (*N* = 289) for *Octopus* species using MEGA v.7 [39], and ii), a subset of those species (N = 46) found along both American coasts using BEAST v.2.3.2 [36]. The tMRCA (time to the Most Recent Common Ancestor) prior to the node parent of identified geminate clades containing 21 sequences of 482 pb length was defined as 3 ± 0.5 million years ago (Mya) [40, 41]; the mutation model [42] selected a strict molecular clock with a starting value of 0.001; trees were obtained with a Yule-type speciation process, with clock rate estimated using BEAST, performing a 50 x 10^6^ MCMC chain length. Demographic dynamics were investigated under the distribution of pairwise differences in mismatch distributions [43] and the Bayesian criterion with Bayesian skyline [44].

## Results

### Genetic diversity

NEA *O*. *vulgaris* samples were from four insular localities (the Azores, Madeira, Canaries, and Cabo Verde oceanic archipelagos), and two off the continent (Galicia and Atlantic Morocco). These samples provide an appropriate mtDNA sequence dataset to appraise Macaronesian phylogeographic scenarios, and to evaluate taxonomic homogeneity within *O*. *vulgaris* (*s*.*s*.) [7].

Control region and COX1 sequences were submitted to GenBank and are available with accession numbers MN704980–MN705275, and MN705276–MN705317, respectively.

Mitochondrial control region alignment included 296 sequences of 637 bp length, ranging from 631 to 632 bp (given a single 1bp indel). As expected for this segment, the base proportion was highly skewed towards AT (82.35%), with polymorphic sites accounting for 10.5% of sequence length. Three specimens, from Azores (N = 2) and Canaries (N = 1), had a 63 bp duplication starting at position 144, which was removed from the final alignment. A total of 64 haplotypes were detected, with a haplotype diversity (*h*) of 0.8783, and a nucleotide diversity (π) of 0.02039. At the population-level, diversity estimates varied, with *h* ranging 0.653 to 0. 8771, and π 0.002 to 0.02, depending on the presence or absence of highly divergent haplotypes within a population (Table 1).

The only negative Tajima’s *D* values, expected for population expansions, were obtained from the Galicia and Cabo Verde samples, which also had significant Fu’s *F*_S_ values and lower π and τ estimates. The Cabo Verde sample had the highest haplotype diversity (*h* = 0.8771), whereas the lowest values were obtained from the Azores and Canaries samples (*h* = 0.6531 and *h* = 0.7072, respectively). Samples from Galicia and Cabo Verde had the lowest nucleotide diversity (π) values, π = 0.0018 and 0.0049, respectively, and the haplotype set was relatively homogeneous.

The additional alignment of COX1 641 bp length sequences, elaborated from a subsample of 42 individuals which showed different haplotypes at the CR sequence, consisted in only 13 COX1 haplotypes with a nucleotide diversity (π) of 0.0063.

**Fig 2:**
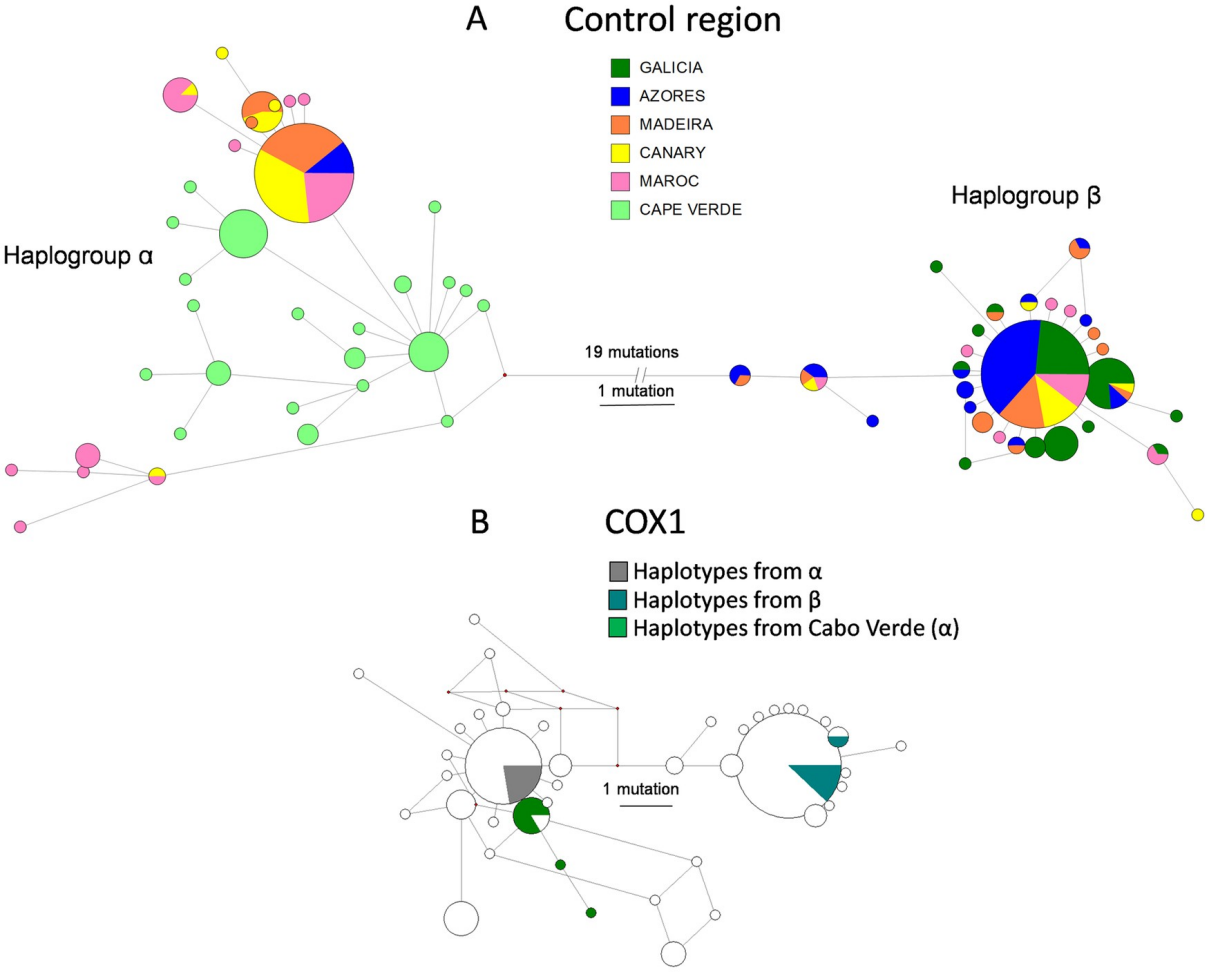
Haplotype network using the median joining algorithm for the *Octopus vulgaris* mitochondrial control region (A), and COX1 (B) DNA sequence alignment. Circles represent single haplotypes, with their area proportional to haplotype frequency. Colors suggest geographic origin (A), or haplogroup affinity (B). Connecting line length is proportional to the number of mutational steps.

The haplotype network built on control region sequences reveals two highly divergent haplogroups (α and β) with a near equal frequency (50.7% and 49.3%, respectively), with 35 and 29 haplotypes, respectively, differentiated by at least 20 mutations (Fig 2A). Within-haplogroup mean distances were 0.006 (α) and 0.002 (β), whereas the between-haplogroup net distance was 0.033 (S.D. = 0.007). A single highly frequent haplotype was detected within each haplogroup, accounting for 43% and 52% of haplotypes in haplogroups α and β, respectively. Haplogroup α had the greatest genetic diversity (*h* = 0.797, π = 0.0058), while haplogroup β (*h* = 0.7124, π = 0.0015) exhibited a typical star-conformation.

The haplogroup structure is also observed, but a lower resolution, from COX 1 sequences data set acquired from this and others studies. A total of 41 Atlantic-Mediterranean haplotypes were mined from GenBank and BOLD repositories, resulting in a consensus alignment containing 58 variable positions. The COX1-based network revealed the two most common and ubiquitous haplotypes, reported from the North Atlantic, including the Mediterranean Sea, to be identical or closely related to those we report (Fig 2B). In contrast with control region data, only 5 mutations occur between these haplotypes, so distance values are lower when considering COX1 subsample sequences, with 0.003 and 0.001 (S.D. = 0.001) values within haplogroups, and 0.008 (S.D. = 0.003) between them.

Haplogroup frequency differs at different sampling sites. Haplogroups also have opposing clinal distributions. In the extreme southern (Cabo Verde) and northern (Galicia) sampling locations, only one haplogroup, α or β, respectively, was detected (Fig 1).

### Population structure

Population differentiation was significant (*P* < 0.05, 10,100 replicates) for the majority of pairwise comparisons. Exceptions involve comparisons between the Madeira, Canaries (*P* = 0.637), and Morocco (*P* = 0.053) samples. However, the Morocco sample had a low, but significant *F*_ST_ value relative to the Canaries sample. Excluding these estimates, the mean *F*_ST_ value, *F*_ST_ = 0.1447, was high. The highest differentiation values were estimated from comparisons involving the Cabo Verde sample (Table 2).

**Table 2:** Population pairwise *F*_ST_ values estimated from the mitochondrial control region.

	Galicia	Azores	Madeira	Canaries	Morocco	Cabo Verde
Galicia						
Azores	0.07989*					
Madeira	0.14193*	0.11913*				
Canaries	0.18820*	0.16347*	-0.00739			
Morocco	0.12521*	0.12365*	0.02039	0.02848*		
Cabo Verde	0.16822*	0.23492*	0.16903*	0.20614*	0.13276*	

* *P*<0.05

**Fig 3:**
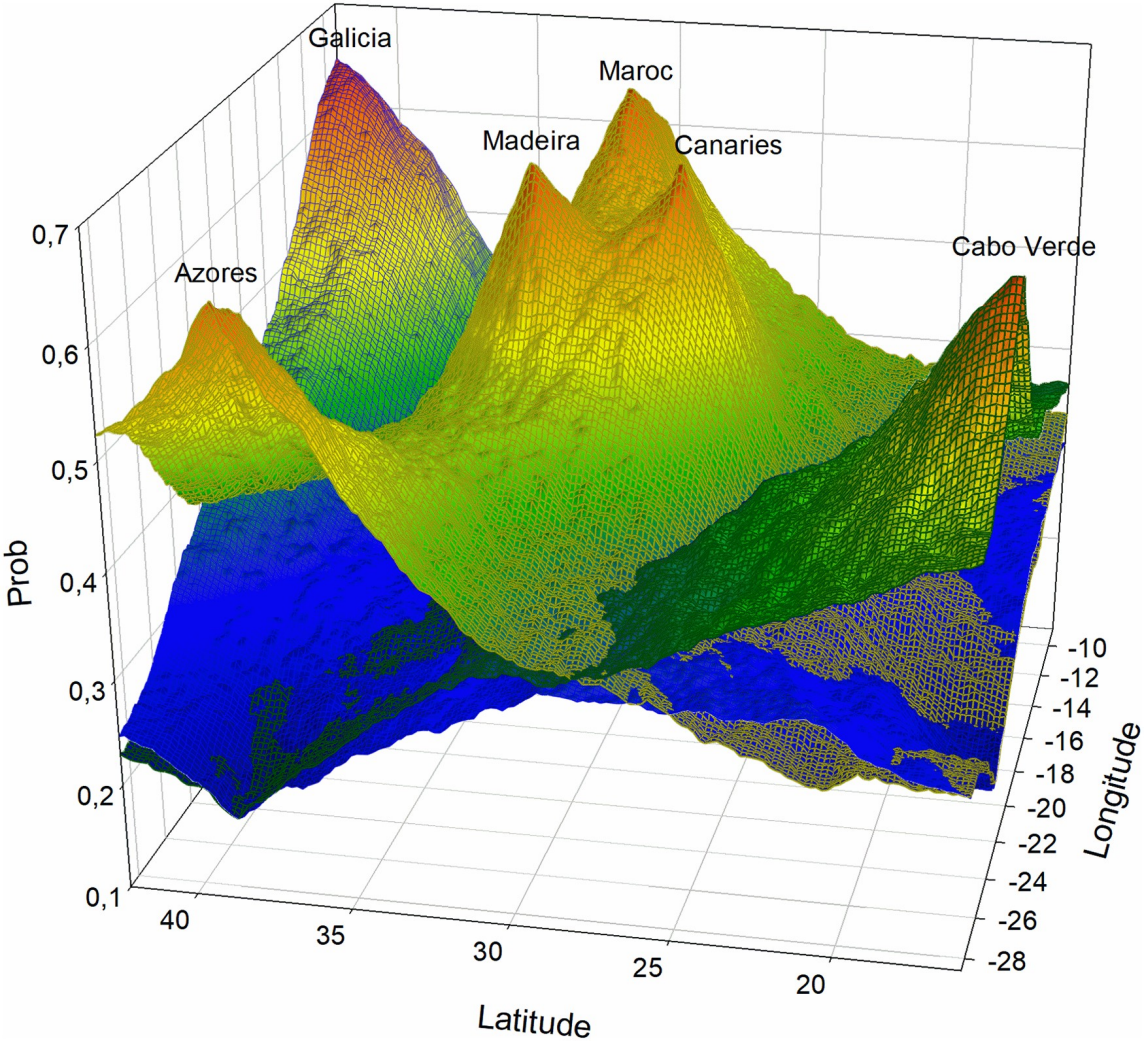
Probabilistic topography for assigning northeastern Atlantic *Octopus vulgaris* to clusters using Geneland. Mesh colors: blue (cluster 1), yellow (cluster 2), green (cluster 3). Probability values are depicted in a gradient from high (red) to low (blue).

Geneland results suggest the existence of three differentiated populations (*k* = 3, 55.62%, average log posterior probability *P* = −233.8595) under the uncorrelated allele frequency model. The correlated allele frequency model produces four populations (*k* = 4), but with reduced support. The three estimated groups include the Galicia sample (cluster 1), the Morocco, Madeira, Canaries and Azores samples (cluster 2), and the Cabo Verde sample (cluster 3). For the less-supported correlated model, cluster 4 includes only the Azores sample. The probability of cluster membership exceeded 0.6 for all samples (Fig 3).

**Fig 4:**
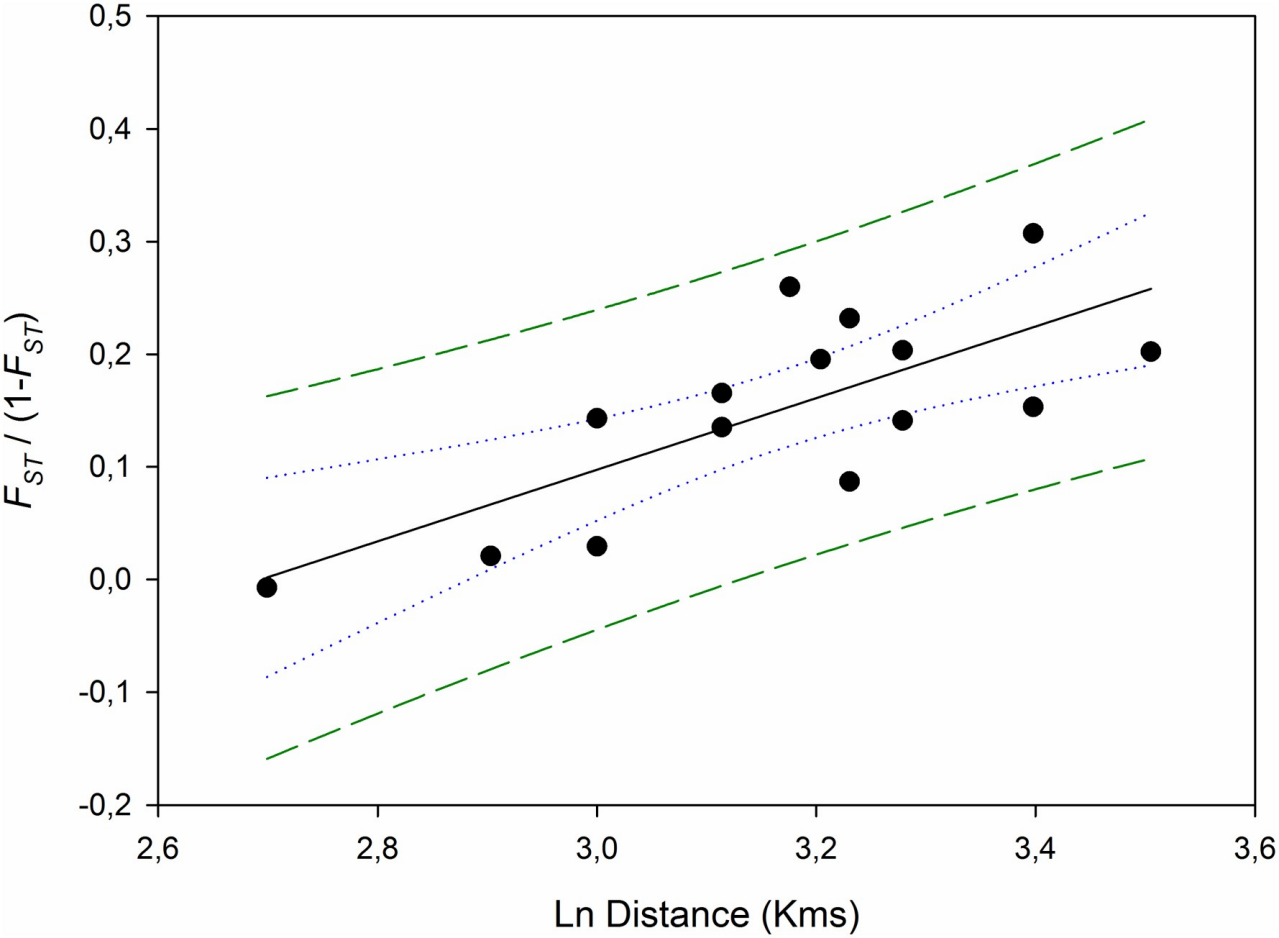
Regression analysis of linearized *F*_ST_ values and geographic distance among *Octopus vulgaris* (*R*^2^ = 0.5482, *P* = 0.0016). Lines depict the 95% confidence (blue-dots) and 95% prediction (green dashes) bands.

A pattern consistent with an isolation-by-distance scenario was observed, resulting in a significant regression between the linearized *F*_ST_ and the Ln of the distance among sampling sites (*R*^2^ = 0.5482, *P* = 0.0016) (Fig 4). A Mantel test [34] revealed a positive correlation between genetic and geographic distances (*Z* = 0.4743, r = 0.7627, *P* ≤ 0.011, 1000 randomizations).

**Fig 5:**
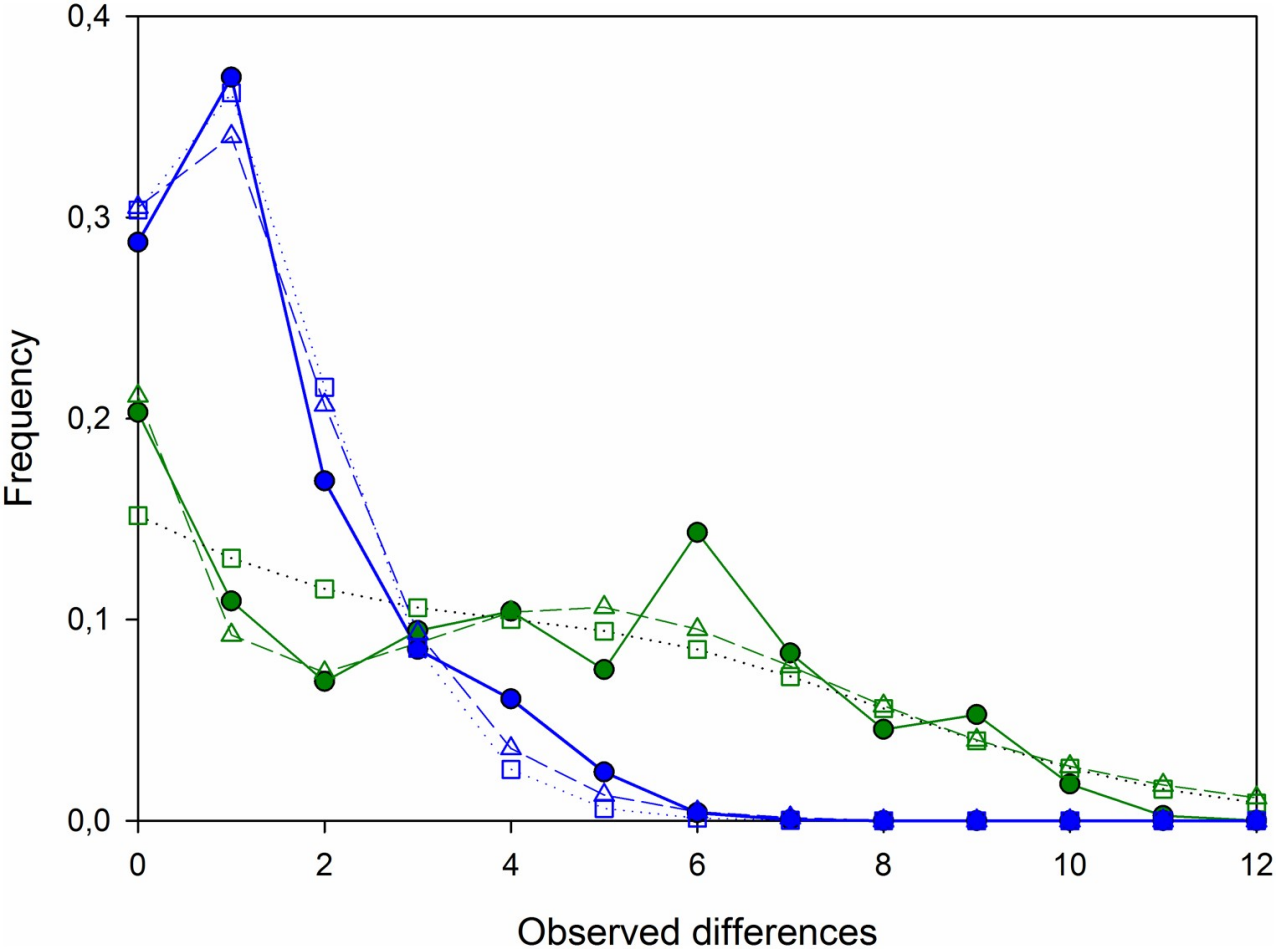
Mismatch distributions of two main haplogroups in northeastern Atlantic *Octopus vulgaris*. Solid lines (green, haplogroup α) and blue (haplogroup β); squares and dotted lines depict a spatial model, and triangles and dashed lines depict a sudden expansion model.

Haplogroup-specific mismatch distributions (MD) had different profiles. The haplogroup α MD was multi-modal, with a distribution that better fitted a spatial than sudden expansion model. In contrast, the haplogroup β MD was unimodal, with a distribution that was similar to both sudden and spatial models (Fig 5). The τ value differed for each haplogroup MD, thus, for the spatial distribution of haplogroup α, τ = 4.114. For haplogroup β, τ = 1.191, consistent with a population undergoing a sudden expansion model, or τ = 0.781, if the spatial model was considered. Although Tajima’s *D* was negative for both haplogroups, it was only significant for the high value for haplogroup β (−2.20338, *P* < 0.0001). Both Fu values were large, negative, and significant (*P* < 0.001).

**Fig 6:**
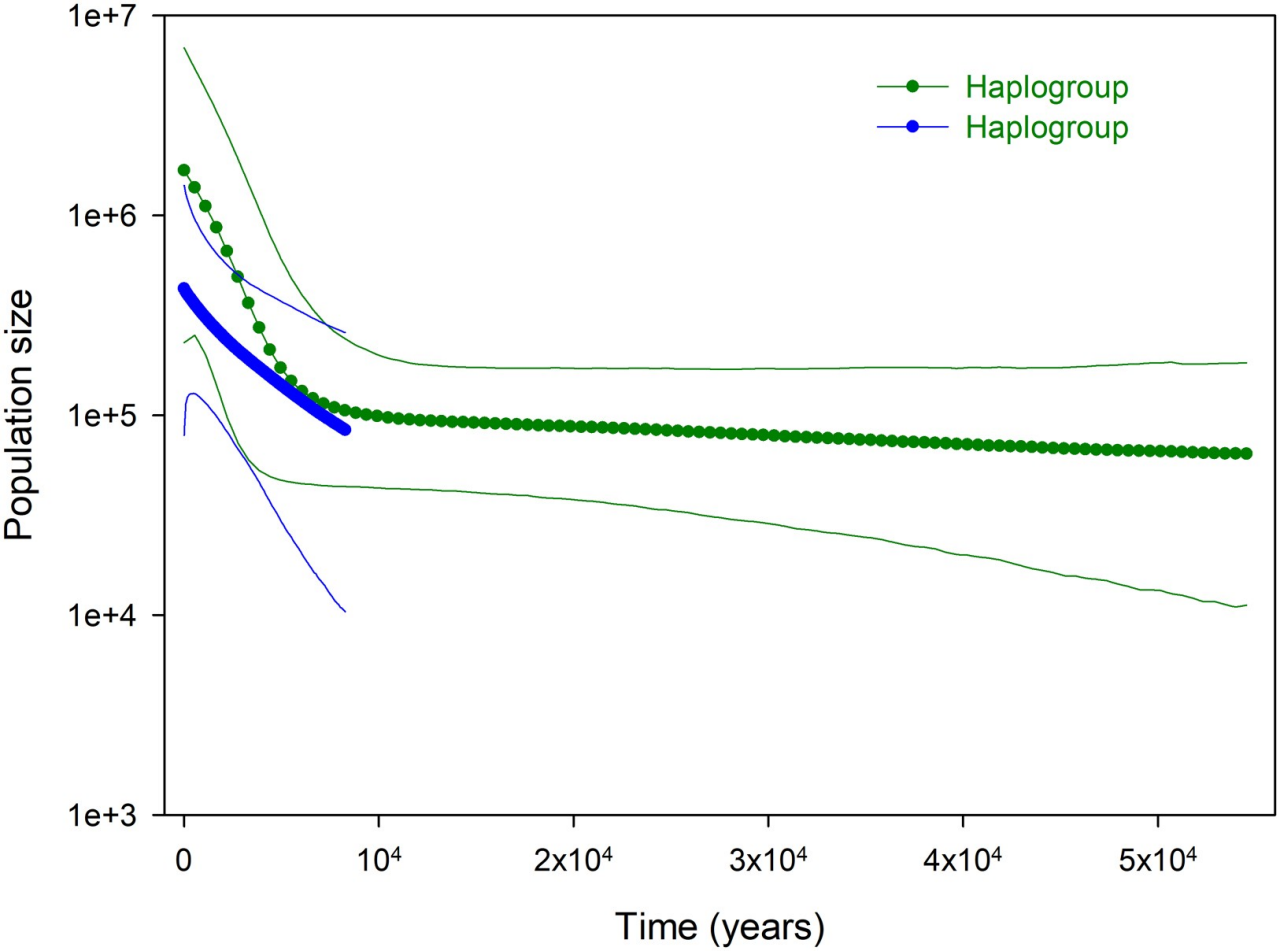
Bayesian skyline plot based on mitochondrial control region coalescent analysis for *Octopus vulgaris* haplogroups in the northeastern Atlantic.

A Bayesian coalescent analysis suggests haplogroup-specific demographic patterns. Haplogroup α had a skyline plot, resulting from a stable population from 50 Kybp (thousand years before present) to 10 Kybp, after which the population continued to grow in size. This increment is near parallel to that observed for haplogroup β, also near 10 Kybp. The estimated effective population size is slightly higher for the older and genetically diverse haplogroup α (Fig 6).

### Dating

**Fig 7:**
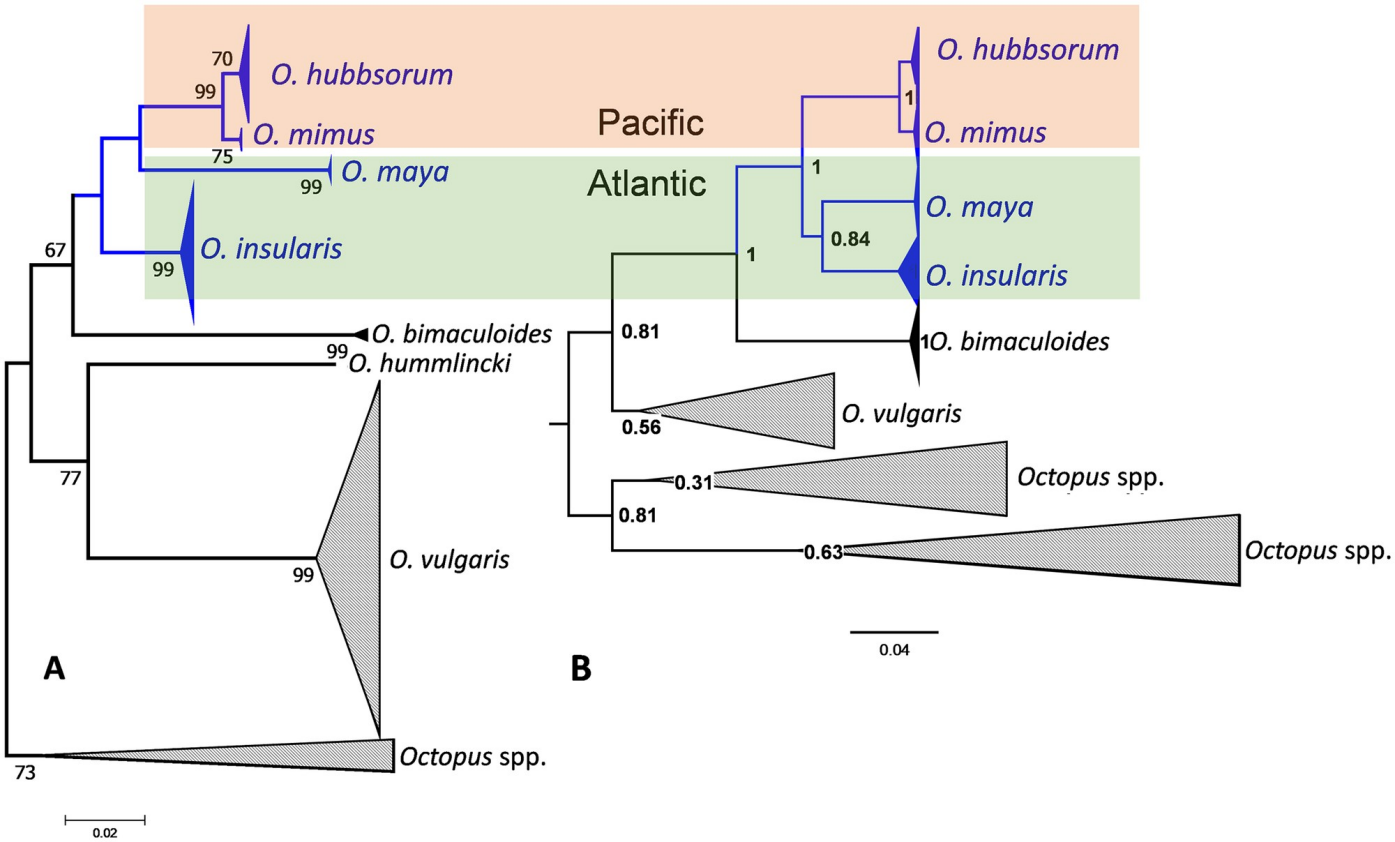
Phylogenetic relationships among *Octopus* species with distributions including the Panama Isthmus, and suggested timeframe for molecular rate calibration. A. Phylogenetic tree based on Tamura-Nei distances from 289 COX 1 gene sequences of 474 bp length, inferred using the Neighbor-Joining method with a bootstrap test (2000 replicates) conducted in MEGA7. B. Phylogenetic tree estimated by Bayesian inference using a subset of 46 COX1 sequences of 482bp length, using BEAST. Trees are midpoint rooted.

The reconstructed phylogenetic relationships of *Octopus* species on both sides of the Panama Isthmus, based on all available COX1 data, suggests that *O*. *insularis*, *O*. *maya* (Voss & Solis, 1966), *O hubbsorum* (Berry, 1953), and *O*. *mimus* (Gould, 1852) have diverged the least, and are involved in a putative geminate relationship (Fig 7A). When phylogenetic relationships focus on these species, a sister clade relationship for two pairs of trans-isthmian geminate species can be inferred: 1) a Pacific clade (*O*. *mimus* and *O*. *hubbsorum*) with 99% bootstrap support (species considered synonyms by Pliego-Cárdenas et al. [45], and 2), an Atlantic sister clade (*O*. *insularis* and *O*. *maya*). In a basal position is *O*. *vulgaris*, while *O*. *bimaculoides* (Pickford & McConnaughey, 1949) holds a divergent position (Fig 7B). The mean *p*-distance between the geminate Atlantic pair (*O*. *insularis* and *O*. *maya*) and Pacific clade (*O*. *mimus*/*hubbsorum*) was 0.06. We suggest approximate mutation rates of 0.0086 and 0.012 mutations per site per million years (My) using estimated dates of Panama Isthmus closure of 3.5 Mya [40], and 2.5 Mya [41], respectively. When this rate was estimated by Bayesian inference, using the same Pacific and Atlantic geminate clades, a mutation rate value of 0.0154 (S.D. = 0.00004) substitutions per site per My was estimated for the COX1 gene, which is close to the mean estimated rate for Protostomia [46], and within the range of mutation rates for the cuttlefish *Sepia officinalis* Linnaeus, 1758 [47] and Humboldt squid *Dosidicus gigas* d’Orbigny, 1835 [48].

A direct, specific and reliable estimate of *Octopus* control region mutation rate was unavailable for coalescence dating of haplogroups, so we indirectly approximated this from COX1 calculations. Relationships between COX1 and CR mutation rates were obtained by estimations from respective homologous data sets. The ratio between each gene-specific ϑ (ϑ = 2 *Nμ*, *N* = effective population size, *μ* = mutation rate) equals the ratio of mutation rates between COX1- and CR-linked loci, as *N* is identical for the same sample [3]. Thus, ϑ values were 3.486, and 12.317, for ϑ _COX1_, and ϑ _CR_, respectively. The mutation rate for the CR (μ _CR_) was 3.53 times that of COX1(μ _COX1_). Based on this COX1 calibration, our μ _CR_ estimate was 5.44 x 10^−8^ per site per year.

Dating of demographic or spatial expansions was obtained by *t* = τ / (2 μ_CR_) with a generation time of 0.57 years [13–16, 18]. Haplogroup α had a mismatched distribution fitting a spatial expansion model, resulting in a 59.4 Kya (thousand years ago), since that expansion. For haplogroup β, dating was 11.3 Kya for the spatial, and 17.2 Kya for the demographic expansion model.

Haplogroup coalescence BSL dating agrees with expansion estimates from mismatch distributions. Haplogroup α was traced to about 50–60 Kya, whereas the coalescence of haplogroup β occurred more recently (8–11 Kya), after the last glacial maxima (LGM). It is during the recent period that both haplogroups experienced drastic increases in population size.

## Discussion

### Genetic diversity

Overall diversity values, *h* (0.8783) and particularly π values (0.0204), are indicative of mixing of historically isolated lineages, herein referred to as haplogroups α and β. When calculated separately, the *h* and π value of each haplogroup decreases significantly (mean: *h* = 0.7547, π = 0.0037). A global sample of the giant squid, *Architeuthis dux* (Steenstrup, 1847) (N = 43) has both lower control region haplotype (*h* = 0.613) and nucleotide (π = 0.0017) diversity [49]. These global values are almost identical to those estimated for certain sampling sites in our study (Table 1). For example, samples from Galicia (*h* = 0.786, π = 0.0018) and Pico (*h* = 0.653, π = 0.0097) are similar to values for *A*. *dux*, indicative of exceptionally low global genetic diversity [49]. For COX1 data, the nucleotide diversity (π) value of 0.0063 is similar to that for *O*. *vulgaris* (0.004) in the Atlantic [50]. Population-level values for *h* and π are consistent with general patterns for animal COX1 diversity (*h* = 0.7013, π = 0.0036) [51], but higher than values reported for other cephalopods [52].

The mean COX1 divergence between haplogroups (1.0%) is an overestimate because it is calculated on sequence data from selected specimens harboring different CR haplotypes. The highest COX1 *p*-distance, 1.6%, is unremarkable (typical of intraspecific values), lower than the estimate between eastern and western Atlantic *O*. *vulgaris* (2.6%) of Sales et al. [50], and similar to the maximum estimated divergence value (1.3%) for South African populations [28]. In contrast, the mean value between two haplogroups for *Enteroctopus dofleini* Wülker, 1910, 2.9%, suggests cryptic lineage speciation [53]. Considering the >10-fold mean difference between intra (0.08%–0.77%) and interspecific (mean = 2.2%; 0.7%–4.2%) COX1 values in *Pareledone* [54], the *Octopus* haplogroups reported herein likely represent intraspecific lineages.

Different Tajima’s *D* and Fu’s *F*_*S*_ estimated values are not always informative with regard to population size dynamics. Our values are affected by the mixing of divergent haplogroups within most populations, except for those from Galicia and Cabo Verde, where values are negative (as would be expected for a recent expansion event). Otherwise *D* and *F*_ST_ values were positive, indicative of bottleneck events. However, these positive values result from increased nucleotide diversity relative to the number of segregating sites, and in a deficiency of alleles, related to the partitioning of sample sequences into divergent haplogroups. Considering each haplogroup separately, *D* and *F*_ST_ values are negative (more so for β), as expected from an expansion population process.

The total mitochondrial evidence for *O*. *vulgaris*, the COX1 and control region data from our and mined data, suggests two main divergent haplogroups exist throughout the eastern Atlantic. While population structure is complex, haplogroup β has a distribution focused in northern waters, while haplogroup α is primarily southern; both occur in the Mediterranean Sea. The southernmost-detected private haplotypes occur in haplogroup α from Cabo Verde and South Africa samples [28].

The near identical (single mutation) COX1 haplotypes in South Africa [28], Cabo Verde, Canaries, and Atlantic Morocco, low divergence among haplotypes within haplogroup α, and the ubiquity of haplogroup α along the northeastern Atlantic, do not support the existence of cryptic speciation in *O*. *vulgaris* ‘Type I’ as proposed by Norman et al. [7] for the South African coast.

Environmental adaptive patterns in haplogroup distributions have been described for invertebrates [55]. However, the extensive geographical range occupied by haplogroup α, from temperate to tropical Macaronesian waters (32°N) to South Africa (35°S), suggests haplogroup frequency is not correlated with environmental variables. The observed haplogroup frequency gradient is, however, consistent with phylogeographic processes.

### Population structure

The deep open ocean surrounding insular habitats presents barriers to dispersal-driven population differentiation in octopuses [56]. We describe strong population structure along the northeastern Atlantic coast, except for samples from neighboring sites in the Macaronesia central core. These samples from the closest locations to the Madeira and Canaries islands, and continental Moroccan coast, are < 500 km apart, and under the common influence of the southward Canary Current, oceanographic gyres and filaments, and other regional hydrographic structures that might modulate gene flow within this area [3, 4].

Samples from Madeira and the Canaries belong to a single population unit. The distance between them (about 450 km) exceeds the 200 km considered sufficient for promoting genetic differentiation in Iberian coastal populations of *O*. *vulgaris*, when estimated from microsatellite loci [24]. However, intermediate islands (the Desertas and Salvagens) and seamounts could function as stepping-stone habitats, facilitating dispersal [2]. The limited insular habitat, and varied potential for dispersal caused by variable oceanographic conditions in different areas, but also methodological resolution, likely contributes to differences in distances required for genetic differentiation.

The Macaronesia core diverged slightly from the continental Moroccan coast and northern Azores archipelago. Isolation of the Azores population from other Macaronesia archipelagos is a likely outcome of the unidirectional flow of the Azores Current [57], explaining the low frequency of haplogroup α and prevalence of haplogroup β at more northern latitudes. Diverse genetic patterns have been described for Azorean marine invertebrates: the Azorean limpet, *Patella candei* (d’Orbigny, 1840), is highly divergent from conspecific Macaronesian populations [58]; populations of *Lasaea* clams [59] and the barnacle *Chthamalus stellatus* (Poli, 1791) at the Azores also differ from those of other Macaronesian islands [60]. The Azorean barnacle, *Megabalanus azoricus* (Pilsbry, 1916), is, in contrast, genetically similar to the Madeira and Canaries populations [3].

The strong genetic divergence of the Cabo Verde sample is consistent with a recurrent pattern observed for other marine invertebrate populations [3–5, 61]. The biogeographic divergence and composite biotic affinities of this region [62] are supported by a diverse climate and various oceanographic mechanisms that function as barriers, such as the western displacement of the southward Canary Current and the Cabo Verde Frontal Zone [63].

The isolation-by-distance model accounts for observed divergence in *O*. *vulgaris*, and its relationship with geographic distance among sample locations, as reported for continental populations [24, 25]. The most-distant samples from the continental coast of the Iberian Peninsula, the isolated Azorean archipelago, and the southernmost Cabo Verde archipelago, have the highest interpopulation differentiation values to neighboring populations.

Results suggest a biogeographically congruent population genetic structure in the northeastern Atlantic, with three well-differentiated groups, including Macaronesia, northern continental Iberia, and southern Cabo Verde. Within the Macaronesia group, low, but significant levels of differentiation were present in the Macaronesian core (Madeira and Canaries archipelagos), and the more marginal northern Azores archipelago and continental coast of Morocco.

High degrees of genetic differentiation are consistent with short life cycles, phylopatric behavior, progeny care, sedentary lives, and direct development and low dispersal capability of benthic octopus paralarvae [7]. These features all limit the dispersal capabilities of these paralarvae. We propose that asymmetric age expectation (male longevity exceeding that of the female), and dispersal potential promote genetic differentiation. Multiple paternity has been demonstrated for *O*. *vulgaris* [12]; the female, transferring mitochondrial genetic variation, dies shortly after eggs hatch. In a putative scenario for adult migration, the low effective number of females, and the low probability of a female migrating, increase genetic differentiation in this species at mitochondrial loci.

### Phylogeography

The matched dating of demographic or spatial expansions, and coalescence time estimates for the divergent haplogroups α and β, reinforce the role of major, recent climate events in shaping the evolutionary history of northeastern Atlantic *O*. *vulgaris*. Haplogroup α was traced back to around 50–60 Kya, whereas coalescence of haplogroup β occurred more recently, around 8–11 Kya. Both estimates are framed in the context of subtropical East Atlantic Macaronesia paleoclimatology, based on local opal maxima records for glacial terminations [64] and global patterns [65]. Thus, the MIS 5e interglacial peak, and the 5c and 5a interstadials of the last interglacial period occurring 130–70 Kya were preceded by the MIS6 glacial maxima (140 Kya) and followed by the weak MIS4 (75–60 Kya) [66]. The current interglacial MS1 started 7–8 Kya ago, after the Heinrinch (17.5 Kya) and Younger Dryas (12.7 Kya) cold events of the LGM [65, 67]. Thus, haplogroup demographic expansions and coalescence estimations are likely associated to restoration of interglacial conditions following glacial termination events.

The “Sea-Level Sensitive” (SLS) dynamic model [68] for marine island biogeography focuses on the impact of sea-level oscillations driven by glacial-interglacial cycles. During prominent glacial maxima, marine littoral habitat was latitudinally reduced and sea level decreased by approximately 60 m, during the 53,69% of the past 150 Kya, compared to present levels (0 ± 20 m) [68]. Sea-level have risen 100 m to present day levels, at the ends of the aforementioned glacial stages (135–129 Kya and 14.5–9 Kya) [67, 69]. Both temporal reference points likely represent the end of periods of haplogroup differentiation driven by refugial isolation and progressive loss of stepping-stone habitat throughout the Macaronesia region [2], preserving insular genetic differentiation.

Throughout the northeastern Atlantic, it has been identified potential LGM marine glacial refugia, including the Macaronesia archipelagos, Iberian Peninsula, and Mediterranean Sea [70]. The southernmost limit of the glacial front, and low associated sea surface temperatures (SST), reached northern Macaronesia during recent Pleistocene glacial maxima [71]. Embryonic development and growth of *O*. *vulgaris* is sensitive to SST, with temperature limits of 7–33°C [11]. Thus, more southern latitudes provided stable glacial refugia for Atlantic octopuses, preserving lineage richness, reflected in high haplotype diversity in the Cabo Verde population.

The recent LGM-related haplogroup β is more frequent in northern latitudes, and absent from the southernmost Cabo Verde. This haplotype has experienced a recent expansion in more acceptable climatic conditions. At the most northern site (Galicia) it is the only detected haplogroup; its low diversity is consistent with an expected outcome of the evolutionary legacy of the ice ages [72]. Haplogroup α, in contrast, has the opposite distribution, with higher frequencies found at more climatically stable southern latitudes, traced back to the MS4 weak glacial maxima. Haplogroup α includes a divergent set of private haplotypes from Cabo Verde that give its oldest coalescence time. Additional haplotypes not sampled in the present work were apparent in the entire COX1 data set (including GenBank sequences), suggesting a deep coalescence dating within the MIS 5 interglacial. Haplogroup α was also detected from COX1 data from the southern Atlantic (Tristan da Cunha, Amsterdam Island and South Africa), and southwestern Indian ocean (Madagascar) [73], reinforcing a wide, southern distribution, and ancestral origin.

Studies dating mitochondrial haplogroups in Macaronesian marine invertebrates have demonstrated synchronized coalescence with comparable glacial/interglacial recent Pleistocene episodes. The Azorean barnacle *M*. *azoricus* most recent haplogroup β sampled in Azores, Madeira, and Canaries [3], and haplogroups of *Pollicipes pollicipes* (Gmelin, 1789) [4], resemble the coalescence timing, structure and dynamics of *O*. *vulgaris* haplogroup α. All roughly date to the interval following the MIS6 glacial maxima, including the MIS5 interglacial. The recent expansion of *O*. *vulgaris* haplogroup β after the LGM seems to be a common pattern among North Atlantic marine taxa [74].

Macaronesian populations occur through the latitudinal midrange of the Atlantic study area, and consistently exhibit a mix of clinally distributed haplogroups α and β. The low level of gene flow among archipelagos and along the continental coast preserves haplogroup ratios. This pattern differs from others resulting from simultaneous vicariant events following glacial events, with secondary contact [70]. We report haplogroups to have different ages, and for their origin to be related to sequential glacial-related events. Thus, haplogroup β (MIS 2 related) is scattered through an established distribution of the ancestral haplogroup α (MS4/MIS6 related) through sequential contact.

Haplotype (*h*) diversities manifest a weak latitudinal (north-south) cline, with highest diversity values at the Cabo Verde population, mainly because of the high proportion of private alleles, indicative of the role of this area for sustaining a refugial population. However, nucleotide (*π*) diversities have higher values within mid-latitudinal populations, reflecting the contribution of divergent haplotypes to α and β haplogroups. The haplotype network constructed from a limited sample focused on the Macaronesia area replicates a network pattern observed from a complete set of available COX1 data from the *O*. *vulgaris* species sampled worldwide (Fig 2). Consequently, the phylogeographic hypothesis relating the origin of both *O*. *vulgaris* haplogroups to the last two glacial maxima is a good starting point for evaluating the purported *O*. *vulgaris* species complex elsewhere.

Fisheries statistics (including artisanal, local, recreational and commercial captures) for *O*. *vulgaris* are compiled with difficulty in the outermost regions of the Macaronesia archipelago [20]. Our results identify the importance of fisheries management by autonomous regional governments, such as the Azores, Madeira and Canaries, for management of insular and genetically differentiated octopus stocks. For Cabo Verdean fisheries, the genetically differentiated *Octopus* stock could be assigned to the Senegal-Gambia (16–12°N) main stock in western Africa (FAO, 2018), but its status and fishery catch remain unknown.
